# Transcriptome and DNA methylome analysis of peripheral blood samples reveals incomplete restoration and transposable element activation after 3-months recovery of COVID-19

**DOI:** 10.3389/fcell.2022.1001558

**Published:** 2022-10-03

**Authors:** Ying Yin, Xiao-zhao Liu, Qing Tian, Yi-xian Fan, Zhen Ye, Tian-qing Meng, Gong-hong Wei, Cheng-liang Xiong, Hong-gang Li, Ximiao He, Li-quan Zhou

**Affiliations:** ^1^ Department of Physiology, School of Basic Medicine, Tongji Medical College, Huazhong University of Science and Technology, Wuhan, Hubei, China; ^2^ Institute of Reproductive Health, Center for Reproductive Medicine, Tongji Medical College, Huazhong University of Science and Technology, Wuhan, Hubei, China; ^3^ Center for Genomics and Proteomics Research, School of Basic Medicine, Tongji Medical College, Huazhong University of Science and Technology, Wuhan, Hubei, China; ^4^ Hubei Key Laboratory of Drug Target Research and Pharmacodynamic Evaluation, Huazhong University of Science and Technology, Wuhan, China; ^5^ MOE Key Laboratory of Metabolism and Molecular Medicine, Department of Biochemistry and Molecular Biology, School of Basic Medical Sciences, Fudan University Shanghai Cancer Center, Shanghai Medical College of Fudan University, Shanghai, China

**Keywords:** SARS-cov-2, recovery, whole-genome bisulfite sequencing, transposable elements, differentially methylated regions

## Abstract

Comprehensive analyses showed that SARS-CoV-2 infection caused COVID-19 and induced strong immune responses and sometimes severe illnesses. However, cellular features of recovered patients and long-term health consequences remain largely unexplored. In this study, we collected peripheral blood samples from nine recovered COVID-19 patients (median age of 36 years old) from Hubei province, China, 3 months after discharge as well as 5 age- and gender-matched healthy controls; and carried out RNA-seq and whole-genome bisulfite sequencing to identify hallmarks of recovered COVID-19 patients. Our analyses showed significant changes both in transcript abundance and DNA methylation of genes and transposable elements (TEs) in recovered COVID-19 patients. We identified 425 upregulated genes, 214 downregulated genes, and 18,516 differentially methylated regions (DMRs) in total. Aberrantly expressed genes and DMRs were found to be associated with immune responses and other related biological processes, implicating prolonged overreaction of the immune system in response to SARS-CoV-2 infection. Notably, a significant amount of TEs was aberrantly activated and their activation was positively correlated with COVID-19 severity. Moreover, differentially methylated TEs may regulate adjacent gene expression as regulatory elements. Those identified transcriptomic and epigenomic signatures define and drive the features of recovered COVID-19 patients, helping determine the risks of long COVID-19, and guiding clinical intervention.

## Introduction

Emerging SARS-CoV-2 coronavirus which causes coronavirus disease 2019 (COVID-19) and results in complicated health issues has expanded rapidly and swept the whole world, threatening global public health (Guan et al., 2020; Huang et al., 2020). Identified key receptors for SARS-CoV-2 infection include ACE2 (Zhou et al., 2020), TMPRSS2 (Hoffmann et al., 2020), and NRP1 (Cantuti-Castelvetri et al., 2020) which are widely expressed in different tissues of the human body. Symptoms of COVID-19 patients include fever, cough, fatigue, headache, diarrhea, and in severe cases even organ failure. Different patients have various symptoms after SARS-CoV-2 infection, and most of them develop mild to moderate illness. Despite intensive multi-omics investigations (Bojkova et al., 2020; Shen et al., 2020; Xiong et al., 2020; Delorey et al., 2021; Wu et al., 2021), the impact of SARS-CoV-2 on the human body and its long-term effect remains largely unexplored. Notably, SARS patients were tracked after the outbreak of SARS at 2003, identifying a significant incidence of sequelae, including pulmonary fibrosis and limited body function (Gomersall et al., 2004). During the current pandemic, COVID-19 patients were reported to suffer from fatigue, sleep difficulties, and anxiety/depression several months after recovery (Chen et al., 2021). Meanwhile, studies on COVID-19 patients showed severely impaired gut microbiota up to 3–6 months after recovery (Chen et al., 2021; Tian et al., 2021). Moreover, pulmonary dysfunction and ‘plasma metabolites’ remain incompletely restored 3 months after recovery (Chen et al., 2021). Understanding the progress of convalescence of COVID-19 patients is therefore valuable for clinical intervention.

Transposable elements (TEs) are mobile DNA elements and comprise about 40% of human genome (Dewannieux et al., 2003). Four major TE classes are long interspersed nuclear elements (LINEs), short interspersed nuclear elements (SINEs), long terminal repeats (LTRs) and DNA transposons. They are involved in many cellular processes, such as: transcriptional regulation (Percharde et al., 2018), chromatin structure organization (Fadloun et al., 2013), development and cell differentiation (Bernardes et al., 2020; Padmanabhan Nair et al., 2021). Retrotransposons are active TEs capable of “copy and paste” themselves into the human genome through RNA intermediates. Well-known retrotransposons include LINEs, SINEs, and LTRs. LINEs are the most common autonomous retrotransposons, and the mobilization activity of SINEs relies on LINEs (Dewannieux et al., 2003; Cordaux and Batzer, 2009). Retrotransposons can cause insertion, deletion, and inversion in the human genome and therefore their increased expression may lead to reduced genome stability (Gilbert et al., 2002; Symer et al., 2002; Newkirk et al., 2017; Malki et al., 2019). We have reported inappropriate upregulation of TEs especially retrotransposons upon SARS-CoV-2 infection in human cell lines and its potential harm including impaired genome stability, enhanced susceptibility of aged people and cancer patients, aberrant expression of retrotransposon-adjacent genes, and induction of inflammation (Yin et al., 2021). Previous in-depth transcriptome analysis revealed aberrant inflammatory responses in COVID-19 patients (Blanco-Melo et al., 2020). Regarding the impact of SARS-CoV-2 on the human body, a recent study using peripheral blood from COVID-19 patients showed impaired transcriptional network and epigenetic profiles which might be useful for targeted treatment and provided promising hallmarks to predict clinical outcomes (Bernardes et al., 2020). However, how SARS-CoV-2 impacts TEs in the human body remains unclear.

**FIGURE 1:**
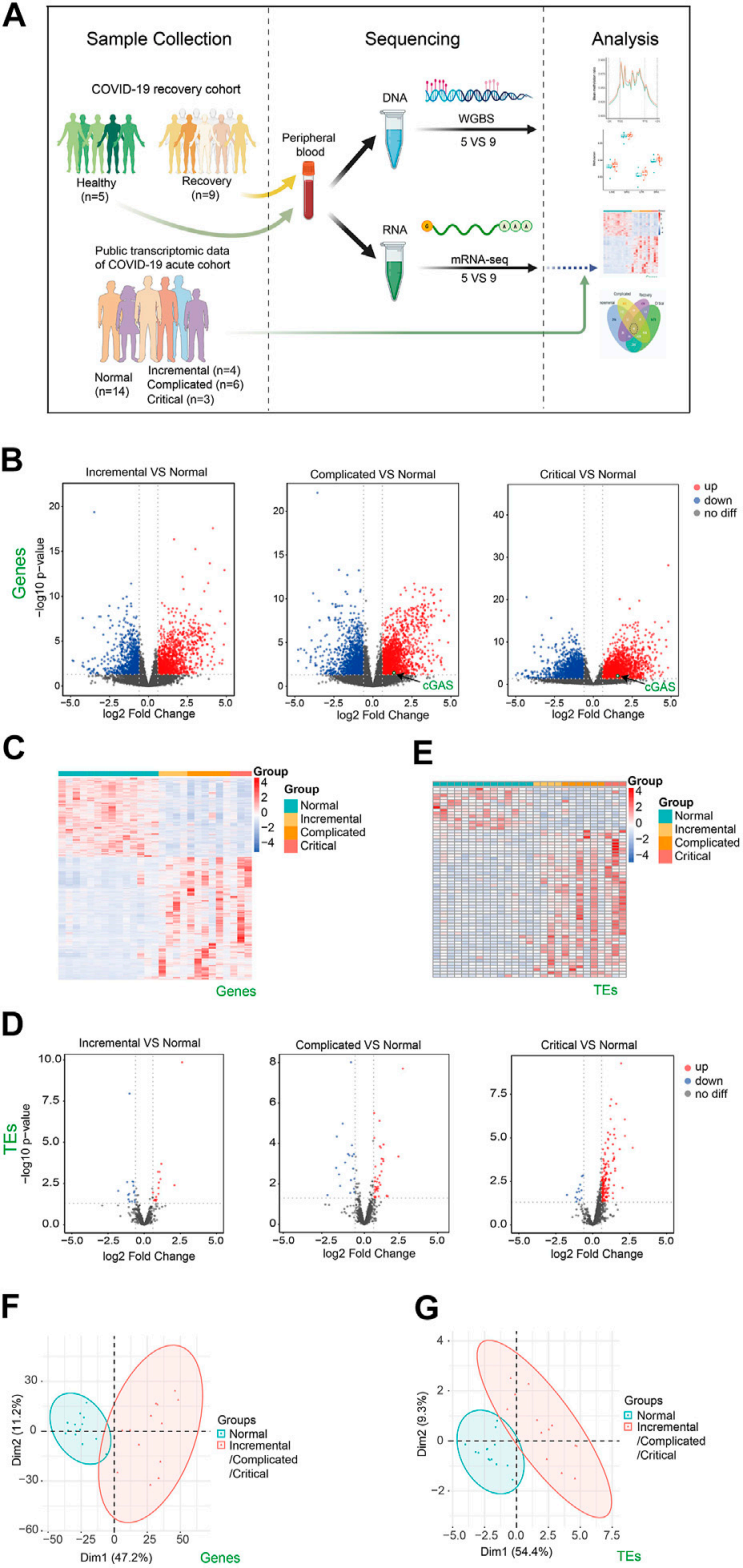
Transcriptome changes of peripheral blood samples from COVID-19 patients at acute phase of incremental, complicated, and critical stages by public RNA-seq data analysis. **(A)** Scheme illustrating our experimental design created with BioRender.com. **(B)** Volcano plots [-log_10_ (*p* value] versus log_2_ (foldchange of gene expression)) displaying transcriptome changes (|log_2_foldchange|>0.6 and *p* value < 0.05) of COVID-19 patients at incremental, complicated, and critical stages. **(C)** Heatmap shows DEGs at incremental, complicated, and critical stages. **(D)** Volcano plots displaying TE expression changes (|log_2_foldchange|>0.6 and *p* value < 0.05) of COVID-19 patients at incremental, complicated, and critical stages. **(E)** Heatmap shows DETEs at incremental, complicated, and critical stages. **(F)** PCA clusters the sequenced samples by normalized counts for DEGs of incremental/complicated/critical group and control group. **(G)** PCA clusters the sequenced samples by normalized counts for DETEs of incremental/complicated/critical group and control group.

Gene expression pattern and epigenetic profile of peripheral blood reflect the whole body’s metabolic status. In the current study, we collected peripheral blood from COVID-19 patients 3 months after recovery from COVID-19 and carried out transcriptome and DNA methylome studies (Figure 1A). Significant amounts of misregulated genes/TEs and differentially methylated regions (DMRs) were identified, indicating incomplete restoration of the human body. Additionally, we identified transcriptome and epigenome signatures which will help identify the long-term impact of COVID-19 on health and provide suggestions for clinical treatment.

## Material and methods

### Patient recruitment and blood sample collection

14 Participants were recruited from the Center for Reproductive Medicine, Tongji Medical College, Huazhong University of Science and Technology. Inclusion criteria for COVID-19 patients included age from 25 to 45 years, having detailed medical records of hospitalization and discharge. Diagnosis of COVID-19 was determined by the New Coronavirus Pneumonia Prevention and Control Program (7th edition) published by the National Health Commission of China (http://www.nhc.gov.cn/xcs/zhengcwj/202003/46c9294a7dfe4cef80dc7f5912eb1989.shtml). Exclusion criteria included asymptomatic cases, taking antibiotics within 2 months, gastrointestinal diseases, and severe basic diseases. Age- and gender-matched healthy controls were recruited during regular physical check-ups in the same hospital with none of them receiving antibiotics within 2 months before collection of blood samples. Ethical approval for the study was obtained by the Ethics Committee for Clinical Research of Reproductive Medicine Center, Tongji Medical College, Huazhong University of Science and Technology. All participants included in the study gave informed consent. 9 male patients in the recovery stage (5 Mild, 4 Moderate) and 5 male healthy controls were recruited for this study. All nine patients meet confirmatory laboratory evidence by detection of SARS-CoV-2 RNA in a clinical specimen using a diagnostic molecular amplification test. No pneumonia symptoms were seen in the imaging of 5 patients with mild symptoms. In 4 patients with moderate symptoms, fever and respiratory tract symptoms can be seen, and pneumonia symptoms were seen on imaging. Age, body mass index (BMI) and other demographic information can be identified in Table 1. Blood (5 ml) was withdrawn from each patient into an Ethylenediamine tetraacetic acid (EDTA)-K2 tube to decelerate blood coagulation. Total RNA was isolated from 2.5 ml blood including PBMC using total RNA isolation kit from SIMGEN. Isolated total RNA and remaining blood samples were subjected to mRNA sequencing and whole-genome bisulfite sequencing (WGBS) by Annoroad Gene Technology Co. Ltd. (Beijing).

**TABLE 1:** Clinical Characteristics of COVID-19 Patients at recovery stage (R1-R9) and Healthy controls (H1-H5) in this study.

No	Gender	Ethnicity	Age (yrs)	Disease severity	Disease duration (days)	Time from discharge to sampling (days)	BMI	CoV-IgM	CoV-IgG
R1	Male	Chinese	31	Mild1	26	70	25.95	+	+
R2	Male	Chinese	29	Mild2	25	79	20.05	+	+
R3	Male	Chinese	24	Moderate1	17	76	28.07	-	+
R4	Male	Chinese	36	Moderate2	13	83	26.23	-	+
R5	Male	Chinese	43	Mild3	12	89	23.66	-	+
R6	Male	Chinese	40	Mild4	32	96	35.43	-	+
R7	Male	Chinese	39	Moderate3	9	98	32.1	-	+
R8	Male	Chinese	36	Moderate4	24	78	26.78	-	+
R9	Male	Chinese	43	Mild5	21	107	25.33	-	+
H1	Male	Chinese	28	Healthy	N/A	N/A	26.4	-	-
H2	Male	Chinese	28	Healthy	N/A	N/A	21.3	-	-
H3	Male	Chinese	32	Healthy	N/A	N/A	21.3	-	-
H4	Male	Chinese	39	Healthy	N/A	N/A	23.0	-	-
H5	Male	Chinese	37	Healthy	N/A	N/A	22.9	-	-

Comparison between COVID-19 group and control group showed no significant differences of age (*p* > 0.05) or BMI (*p* > 0.05). Note that average time from discharge to sampling of COVID-19 patients (R1–R9) was 86 days (about 3 months), and median age was 36 years.

### mRNA isolation for sequencing

Total RNA was used as input material for the RNA sample preparations. Sequencing libraries were generated using NEBNext Ultra RNA Library Prep Kit for Illumina (#E7530L, NEB, United States) following the manufacturer’s recommendations and index codes were added to attribute sequences to each sample. The clustering of the index-coded samples was performed on a cBot cluster generation system using HiSeq PE Cluster Kit v4-cBot-HS (Illumina) according to the manufacturer’s instructions. After cluster generation, the libraries were sequenced on an Illumina HiSeq 2500 system. Approximately 25M of paired-end reads (150 bp ×2) for each sample was generated.

### DNA isolation for methylation profiling

For constructing WGBS libraries, the genomic DNA was fragmented to a mean size of 350 bp, followed by blunt-ending, dA-Tailing, and adaptor ligation. Insert fragments with different sizes were excised from a 2% agarose gel and purified using the QIAquick Gel Extraction Kit (QIAGEN). Purified DNA was bisulfite converted using the EZ DNA Methylation-Gold Kit (#D5006, ZYMO Research, CA, United States) and PCR amplified. The WGBS libraries were sequenced at 20×depth on an Illumina HiSeq 2500 system as paired-end reads (150 bp ×2). Approximately 15–20 × mean coverage was generated for each sample.

### RNA-seq data processing

Raw reads were processed with Trim Galore (v0.6.4) to remove adaptor sequences and poor-quality bases with “--q 20 --phred33 --stringency 5 --length 20 –paired.” To include as many non-uniquely mapped reads as possible, trimmed reads were firstly aligned to the human genome (hg19) by STAR (v2.7.5b) (Dobin et al., 2013) (Dobin, Davis et al., 2013) with default settings including parameters ‘--winAnchorMultimapmax 2000 --outFilterMultimapNmax 1000’. SAMtools (v1.3.1) was used to sort bam files by genomic coordination and make a bam file index. RSEM (v1.2.28) (Li and Dewey, 2011) was used to calculate the FPKM value of genes. TEtranscript (Jin and Hammell, 2018) with default parameters was used to get counts for different transposable elements. UCSC genome browser was used for snapshots of the transcriptome. R package Deseq2 (v1.28.1) (Love et al., 2014) was used to obtain differentially expressed genes (DEGs) and differentially expressed TEs (DETEs). Principal component analysis (PCA) was performed using DESeq2 normalized counts for DEGs/DETEs. Metascape (Zhou et al., 2019) was used to visualize functional profiles of genes and gene clusters. rMATS (v4.1.1) (Shen et al., 2014) was used to identify alternative splicing events with “--readLength 150” and other default parameters. We conducted protein-protein interaction network (PPI network) analysis using STRING database to explore gene interaction at protein level (Szklarczyk et al., 2019). Graphs were created by R. Images were organized by Adobe Illustrator.

### ReMap2022 database for transcription factor binding enrichment analysis

DETEs were transformed to genomic range objects (GRanges) using the GRanges R package. The DETE loci were then compared against the ReMap2022 annotated TFBSs (hg19) for enrichment of specific transcription factors and plotted as enrichment dot plots with the ReMapEnrich R package (Hammal et al., 2022).

### WGBS data processing and quality control

Raw reads were processed with Trim Galore (v0.6.4) to remove adaptor sequences and poor quality bases with “--q 20 --phred33 --stringency 5 --length 20 --paired.” Trimmed reads were then aligned to the human genome (hg19) by Bismark (v0.22.3) (Krueger and Andrews, 2011) using the parameters “-p 6 --parallel 1 -N 0 -L 20 --quiet --un --ambiguous --bam.” SAMtools (v1.3.1) was used to sort bam files by genomic coordination and make a bam file index. PCR duplicates were removed using Picard (v2.23.3). The methylation ratio at each CpG site was constructed using bismark_methylation_extractor model with the parameters “-p --comprehensive --no_overlap --bedgraph –counts --report --cytosine_report --gzip –buffer --size 30G.” For all samples, the average bisulfite conversion success ratio is >99.2%, the alignment ratio is around 80% of read pairs aligning uniquely, and the duplication rate is <5%. For each CpG site, methylation levels were calculated by (methylation reads/total coverage reads). For a more robust analysis, we applied the minimum threshold 3× coverage and also selected CpGs that all samples had their methylation levels. This screening process gave 18 M of CpGs with confident methylation levels. Methylation profiles were calculated by deeptools (v3.5.1) (Ramirez et al., 2014).

### Differentially methylated regions by methylKit

The R package methylKit (v1.14.2) (Akalin et al., 2012) was used to identify DMRs between healthy and recovery groups. The methylation levels at CpG sites were firstly calculated by “methRead” function with mincov = 3. Methylation across the genome was tiled with the ‘tileMethylCounts’ function using the parameters “win.size = 500, step. size = 500, cov. bases = 5”, then ‘unite’ function was used to unite tiled regions with the “destrand = TRUE” parameter. At last, “calculateDiffMeth” function was used to calculate DMRs. DMRs with a minimum of 3 CpG sites and absolute methylation mean difference >10% and q-value < 0.05 were used for further analysis. DMRs were annotated by R package “ChIPseeker” (v1.24.0).

### Statistical methods

Plotting and statistical tests were performed using R (v4.0.2). All statistical tests performed in this study were two-sided. Box plots were generated using the R packages “ggplot2” (v3.3.2) and “ggpubr” (v0.4.0) to show median, first and third quartiles and outliers were shown if outside the 1.5× interquartile range. A two-sided Wilcoxon signed-rank test was used to assess differences between the two groups. Enrichment scores were analyzed using chi-square tests, enrichment score >1 and *p*-value < 0.05 was defined as enrichment.

## Results

### The SARS-cov-2 infection had a profound impact on transcriptome and TE activation which was positively correlated with COVID-19 severity

To identify how the human body responds to SARS-CoV-2, we downloaded and analyzed public RNA-seq data of peripheral blood from COVID-19 patients at acute phase including incremental, complicated, and critical stages, as well as healthy controls (Bernardes et al., 2020) (Supplementary Table S1). A total of 2392, 4,241 and 3,655 misregulated genes were identified in these three stages respectively (Figures 1B,C). DEGs were mainly enriched in immune response, cell cycle, and DNA repair (Supplementary Figures S1A,B). We previously reported that expression of TEs was upregulated upon SARS-CoV-2 infection in human cell lines. This may result in impaired genome stability, increased susceptibility of aged people and cancer patients, misregulation of retrotransposon-adjacent genes, and probably leading to worse clinical outcomes for patients with underlying diseases (Yin et al., 2021). Therefore, we examined the expression of TEs in the acute phase, and observed a gradual upregulation of TEs from incremental (16 upregulated) to complicated (41 upregulated) to critical (164 upregulated) stage (Figures 1D,E; Supplementary Figure S2), indicating that TE expression levels reflected COVID-19 severity. In agreement with the identification of TE activation, we also noticed cGAS upregulation at complicated and critical stages (Figure 1B), while retrotransposon upregulation is one of the major reasons for stimulating the cGAS-STING pathway (Gorbunova et al., 2021). The cGAS-STING pathway drives immune response to cytosolic DNA, activates IFN responses and induces inflammation. Therefore, retrotransposon is a promising therapeutic target for reducing inflammation in COVID-19 patients. Previous study reported release of mitochondrial DNA into the cytoplasm in SARS-CoV2-infected cells, and this may be another reason of activation of cGAS-STING pathway (Domizio et al., 2022). PCA effectively clustered the samples by either DEGs (Figure 1F) or DETEs (Figure 1G). Collectively, our results indicated that SARS-CoV-2 infection triggered immune responses and activated TEs in human peripheral blood.

### Whole-blood transcriptome analysis revealed that SARS-cov-2 impacted the expression of multiple genes and alternative splicing events even after a 3-months recovery

**FIGURE 2:**
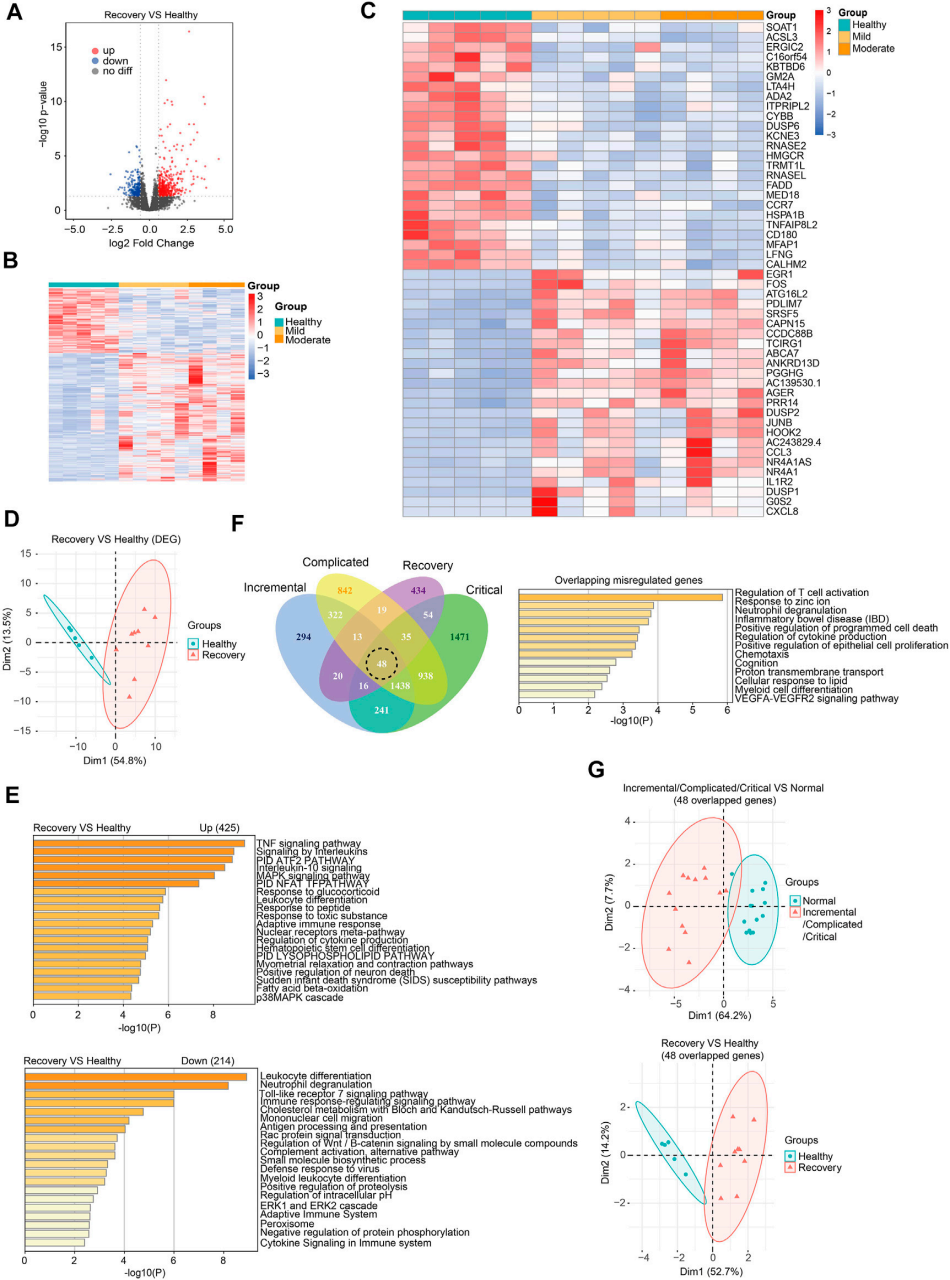
Transcriptome analysis identified gene expression changes of peripheral blood samples from COVID-19 patients after 3-months recovery. **(A)** Volcano plots [-log_10_ (*p* value) *versus* log_2_ (foldchange of gene expression)] displaying transcriptome changes (|log_2_foldchange|>0.6 and *p* value < 0.05) at recovery stage. **(B)** Heatmap shows DEGs in COVID-19 patients at recovery stage. **(C)** Heatmap shows top 25 downregulated and top 25 upregulated genes at recovery stage. **(D)** PCA clusters the sequenced samples by normalized counts for DEGs of recovery group and control group. **(E)** GO analysis of upregulated/downregulated genes for functional enrichment at recovery stage by Metascape. **(F)** Venn diagram identifies 48 overlapping misregulated genes among incremental, complicated, critical stages and recovery stage. GO analysis was further performed to identify functional enrichment of the 48 genes by Metascape. **(G)** PCA of incremental/complicated/critical group and control group (left) and PCA of recovery group and control group (right), using 48 overlapped genes. PCA clusters the sequenced samples by normalized counts for 48 overlapping misregulated genes among incremental, complicated, critical stages and recovery stage.

To understand the recovery progress of the human body after 3-months convalescence from SARS-CoV-2 infection, we recruited COVID-19 patients with mild/moderate symptoms and controls at similar ages in Hubei province, China (Table 1). Generally, patients had a median age of 36 years and the average follow-up time after hospital discharge was 86 days (approximately 3 months). Peripheral blood was collected for subsequent RNA-seq analysis (see Supplementary Table S2 for quality control information). Generally, 425 genes were upregulated and 214 genes were downregulated (Figures 2A–C). PCA effectively clustered the samples by DEGs (Figure 2D). DEGs were enriched in immune response-related biological processes (Figure 2E). We then ask whether the recovery group and acute phase groups share commonly misregulated genes. We found that among 639 misregulated genes in the recovery group, 205 genes were also misregulated in acute phase groups (Figure 2F). There were 48 genes misregulated in all four disease stages and involved in T cell activation and other immune response-related processes (Figures 2F; Supplementary Figure S3). Notably, we found that these genes play various roles in immune responses, and include transmembrane receptors, voltage-gated channel proteins, and transcription factors. As expected, these 48 overlapping misregulated genes can be used to discriminate COVID-19 patients from controls, no matter whether the patients were at acute or recovery stages (Figure 2G), and are therefore useful for clinical diagnosis and treatment of COVID-19.

**FIGURE 3:**
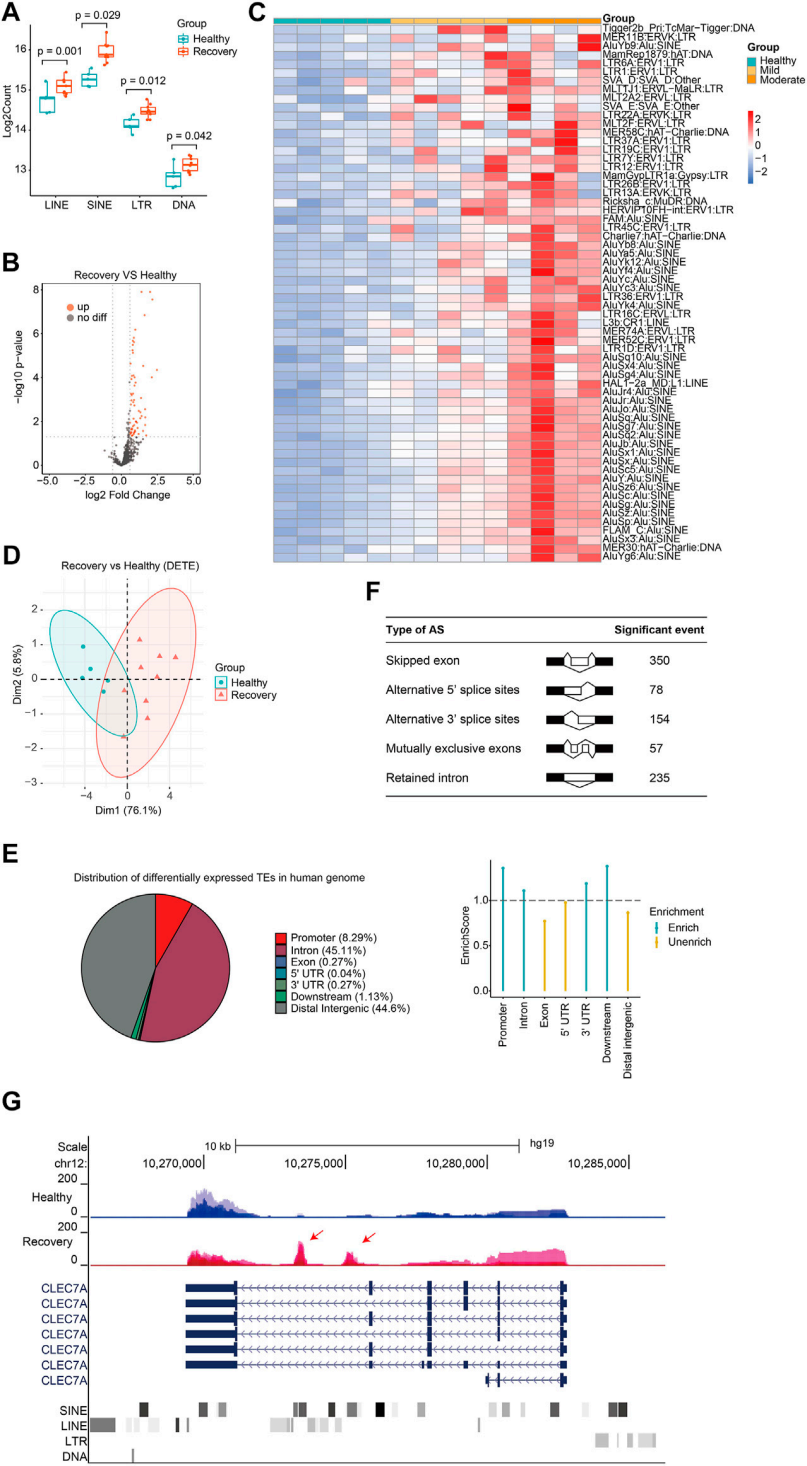
Discovery of aberrantly overexpressed TEs in peripheral blood samples from COVID-19 patients after 3-months recovery. **(A)** Boxplot displaying significantly upregulation of four major TE classes. LINE, SINE, LTR, and DNA transposons are shown, and each dot represents one sample. The median, first, and third quartiles are shown. Two-sided Wilcoxon signed-rank test were used for the comparisons. **(B)**Volcano plots [-log_10_ (*p* value) *versus* log_2_ (foldchange of gene expression)] displaying TE expression changes (|log_2_foldchange|>0.6 and *p* value < 0.05) at recovery stage. **(C)** Heatmap demonstrates all 62 upregulated TEs in COVID-19 patients at recovery stage. **(D)** PCA clusters the sequenced samples by normalized counts for DETEs of recovery group and control group. **(E)** Pie chart shows the distribution of DETE in different gene features of human genome. Enrich score was calculated by (Feature ratio of DETE)/(Feature ratio of TE), chi-square tests was used for statistics, enrichment score >1 and *p*-value < 0.05 was defined as enrichment. **(F)** Change of alternative splicing (AS) events (FDR<0.05) was observed at recovery stage. **(G)** UCSC genome browser view of RNA-seq data demonstrates aberrantly increased TE expression from an intron of CLEC7A gene initiates a novel transcript within CLEC7A gene locus.

Next, we analyzed expression changes of TEs in the recovery group. When we added up all reads for each class of transposon, we observed that global expression of LINE, SINE, LTR, and DNA transposon were all significantly increased (Figure 3A). There were 62 upregulated TEs with most of them belonging to SINE and LTR, while no downregulated TE subfamilies were observed (Figures 3B,C; Supplementary Table S3). Notably, those with moderate illness seemed to have higher upregulation of DETEs than those with mild illness, indicating that TE levels reflected the severity of COVID-19 at the recovery stage. TEs were not randomly upregulated because differentially expressed TEs can well cluster the samples (Figure 3D). Surprisingly, 56 of the 62 upregulated TEs in the recovery group were absent in patients from the acute phase group, indicating that distinct TE subfamilies were activated during the recovery progress.

Different TE subfamilies were misregulated in patients from various stages, and this may be explained by crosstalk among different TE subfamilies and complicated response inside human body. Therefore, we analyzed previously reported transcriptome data of LINE1 knockdown in mouse embryonic stem cells (mESCs) (Percharde et al., 2018) and indeed found abnormally expressed subfamilies like SINE and LTR (Supplementary Figure S4). Another possible reason for different misregulated TE subfamilies between the acute group and recovery group is the diversity of human race and genetic background. Next, we analyzed the distribution of upregulated TEs in the human genome and found that they were mainly enriched in promoter, intron, and downstream regions (Figure 3E). Differentially expressed TEs were overrepresented in the human genome, indicating that upregulation of TEs plays important role in SARS-CoV-2-induced transcriptome changes and serves as a transcriptome signature in COVID-19 patients at the recovery stage. Meanwhile, our transcriptome analysis identified significant changes in alternative splicing events in the recovery group (Figure 3F). Although it is unclear how TEs were activated in the recovery group, increased expression of some of these may induce the generation of novel transcripts inside gene loci and impact alternative splicing patterns (Figure 3G). Beyond transcriptional disturbance, enhanced TE expression may also reduce genome stability, induce inflammation, and cause age-associated disorders.

**FIGURE 4:**
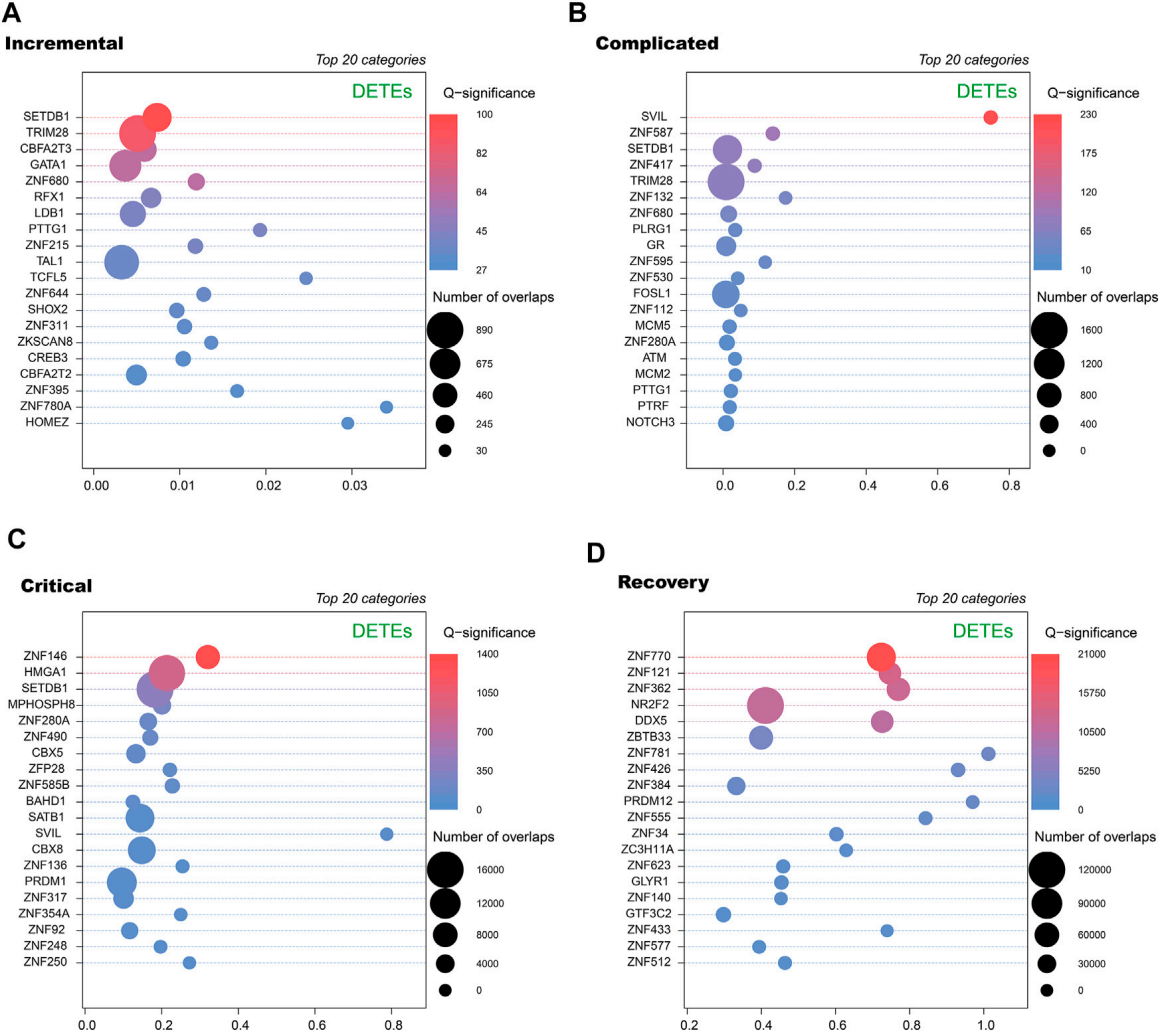
Transcription factors enriched at DETE loci at different stages of COVID-19 patients by ReMap 2022. Dot plots of enrichment of transcription factor binding sites derived from ReMap2022 ChIP-seq database at DETE loci within the human genome in incremental **(A)**, complicated **(B)**, critical **(C)** and recovery **(D)** groups by ReMapEnrich R package.

To further annotate DETEs, we used ReMap2022 to obtain transcription factor binding sites (TFBSs) from publicly available ChIP-seq dataset (Hammal et al., 2022). By comparing loci of the DETEs with annotated TFBSs, we observed distinct categories of transcription factors enriched at TE loci in different stages (Figure 4). Notably, zinc finger proteins (ZNFs) are key regulators of TE expression, and are frequently identified in enriched transcription factors but diverse ZNFs are seen in these stages. Therefore, alteration of DETEs at acute and recovery stages may be caused by dynamic expression of ZNFs at these stages.

### Whole-blood DNA methylome analysis of the recovery group identified genome-wide DMRs which mainly localized at TEs’ loci between healthy and recovery groups

Gene expression can be regulated by DNA methylation which can be long-term memorized. Thus, we next examined the expression of DNA Methyltransferase (DNMT) and Ten-eleven Translocation (TET) family genes which play key roles in controlling DNA methylation status. Almost all of these genes showed altered expression levels in patients at the recovery stage (Supplementary Figure S5). This prompted us to further investigate changes of in genome-wide DNA methylation in COVID-19 patients after a 3-months recovery.

**FIGURE 5:**
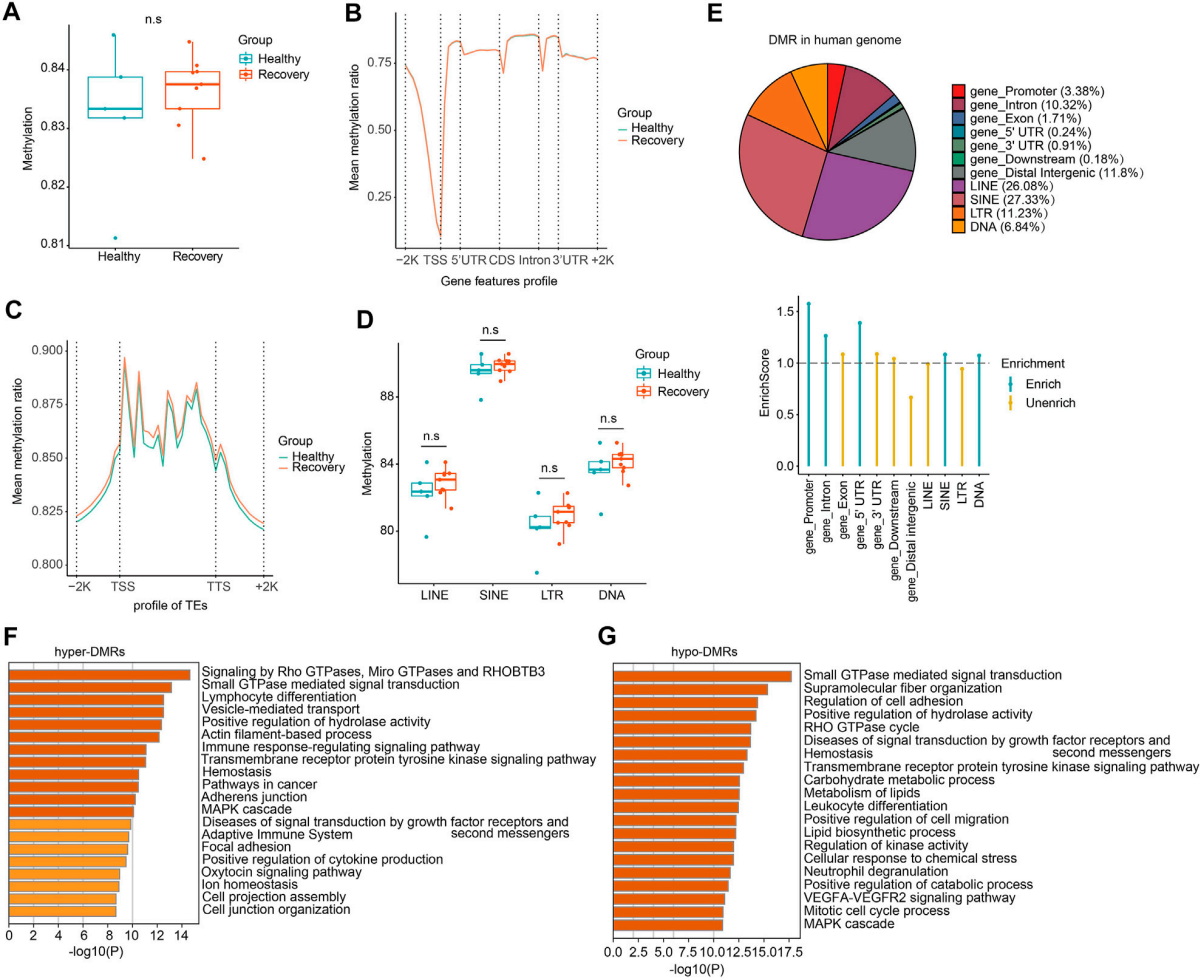
WGBS analysis of recovery and control group revealed comparable DNA methylation profiles and genome-wide DMRs which mainly localized at TE loci. **(A)** The whole mean methylation level per sample of 18M CpG sites for Healthy and Recovery group, and each dot represents one sample. Boxplot shows the mean methylation status per samples of Healthy and Recovery group, each dot represents mean methylation status of one sample. **(B)** Methylation profile of gene features per sample. Methylation levels were measured in each 200 bp interval of a 2 kb region upstream and downstream of all annotated genes. Methylation was measured in 10 equally sized bins for CDSs and introns, and 5 equally sized bins for UTRs. **(C)** Methylation profile of TEs per sample. Methylation levels were measured in each 200 bp interval of a 2 kb region upstream and downstream of all annotated TEs, then for each TEs methylation levels were measured in 20 equally sized bins. **(D)** Boxplot of mean methylation status of four major TE classes. LINE, SINE, LTR, and DNA transposons are shown, and each dot represents one sample. **(E)** Pie chart demonstrates distribution of DMRs in different gene features of human genome and TE subfamilies. Enrich score was calculated by (Feature ratio of DMR)/(Feature ratio of reference genome), sliding windows was made across reference genome with 500 bps consistent with our call DMR strategy. Chi-square tests was used for statistics, enrichment score >1 and *p*-value < 0.05 was defined as enrichment. **(F)** Functional enrichment of genes with hyper-DMRs identified at promoter/gene body by Metascape. **(G)** Functional enrichment of genes with hypo-DMRs identified at promoter/gene body by Metascape. For above boxplots, the median, first, and third quartiles are shown. Two-sided Wilcoxon signed-rank test were used for all the comparisons.

To study how DNA methylome was changed between healthy and recovery groups and how it may correlate with transcriptome alteration, we examined whole-blood DNA methylome by WGBS (see Supplementary Table S4 for quality control information). Generally, whole-genome CG methylation levels showed no significant alterations (Figure 5A). Analysis of genomic regions from 2 kb upstream of transcriptional start sites (TSSs) to 2 kb downstream of transcriptional end sites (TESs) indicated no significant CG methylation changes (Figure 5B). Next, we examined loci of TEs and identified minimum alteration of global CG methylation level (Figures 5C,D).

To explore changes in CG methylation patterns between healthy and recovery groups, we analyzed DMRs and identified 18,516 DMRs in total (absolute methylation mean difference >10% and q-value < 0.05). 8,724 DMRs had hypo-methylation (hypo-DMRs) and 9,792 DMRs had hyper-methylation (hyper-DMRs) in the recovery group. Identified DMRs were mainly enriched at gene promoter, intron, and certain TE regions (Figure 5E). Although we didn't detect significant effects of COVID-19 on DNA methylome in genome-wide analysis, GO analysis showed that hyper-DMRs and hypo-DMRs in gene promoter/body regions were involved in various signal pathways, immune response, and metabolism (Figures 5F,G).

**FIGURE 6:**
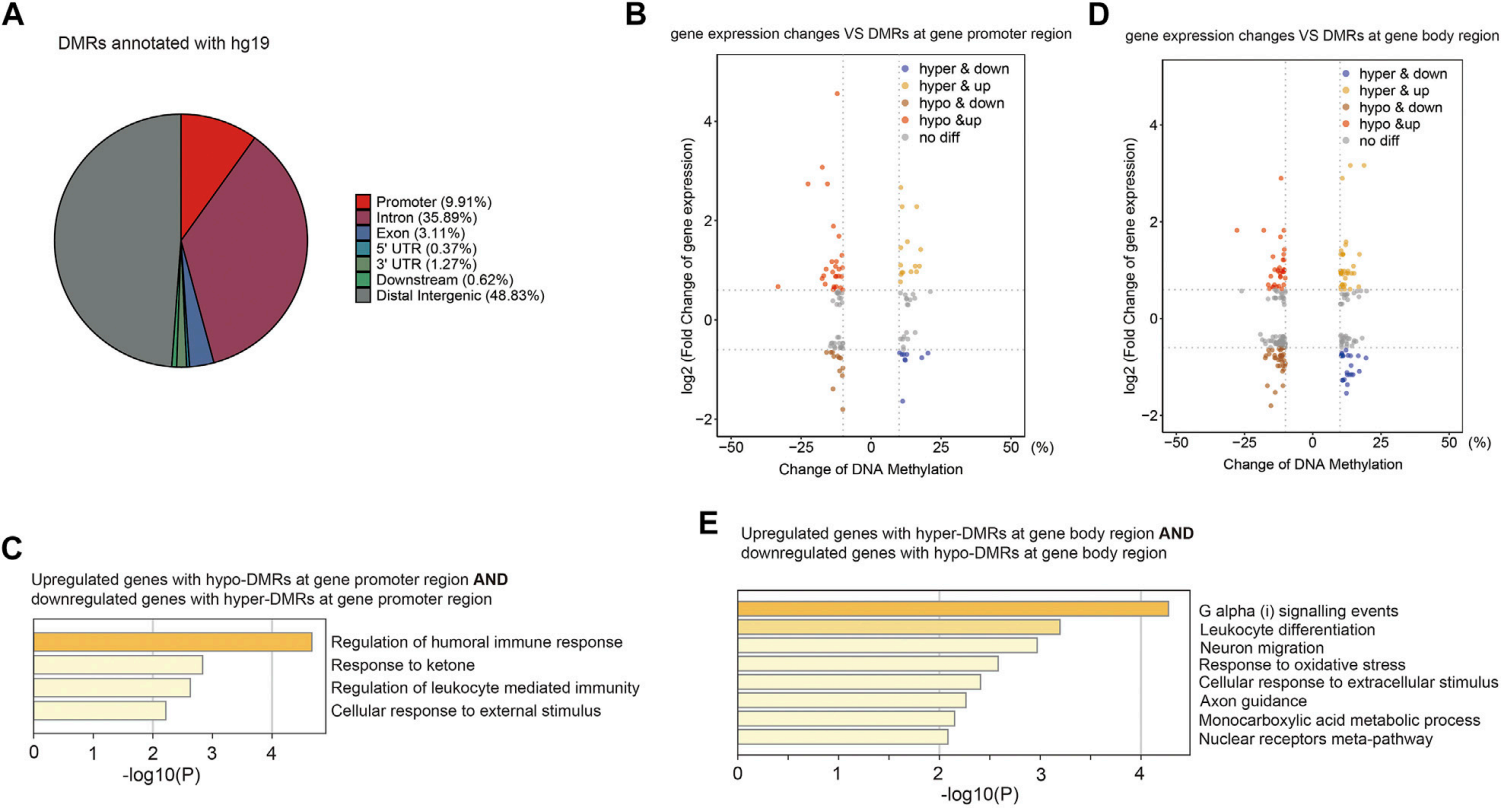
Aberrant DNA methylation at gene loci may be involved in immune response-related gene misregulation. **(A)** Pie chart (without annotation of TEs) demonstrates distribution of DMRs in human genome. **(B)** Scatter plot of the relationship between DNA methylation differences at gene promoter and their expression differences. **(C)** Functional enrichment of upregulated/downregulated genes with hypo/hyper-DMRs in gene promoter regions by Metascape. **(D)** Scatter plot of the relationship between DNA methylation differences at gene body and their expression differences. **(E)** Functional enrichment of upregulated/downregulated genes with hypo/hyper-DMRs in gene body regions by Metascape.

CG methylation at different genomic loci may play different roles. Without annotation of TE loci, the distribution of identified DMRs in the human genome was as follows: 9.91% in promoters, 40.64% in gene bodies, 49.45% in intergenic and other regions (Figure 6A). We then examined expression levels of identified genes with DMRs at promoters or gene bodies. We found that nine hyper-DMRs at promoters were associated with downregulated genes, while 28 hypo-DMRs at promoters were associated with upregulated genes (Figure 6B; Supplementary Table S5), and these genes were mainly involved in immune responses (Figure 6C). Meanwhile, 27 hypo-DMRs at gene bodies were associated with gene downregulation, and 13 hyper-DMRs at gene bodies were associated with gene upregulation (Figure 6D), mainly involved in stress response and related signaling pathways (Figure 6E).

**FIGURE 7:**
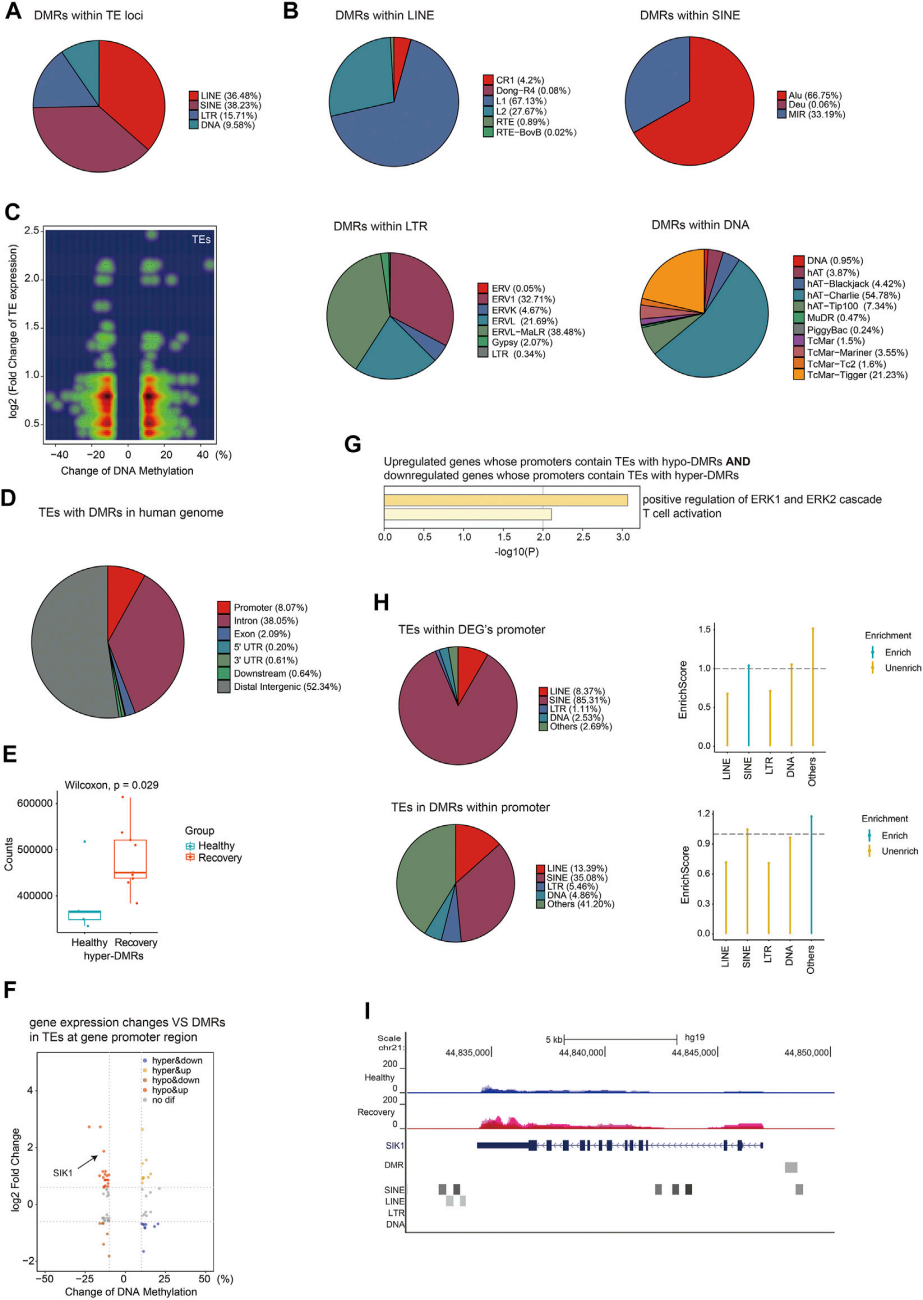
Aberrant DNA methylation at TE loci may be involved in gene regulation. **(A)** Pie chart demonstrates distribution of DMRs in TE subfamilies. **(B)** Pie chart demonstrates distribution of DMRs in LINE/SINE/LTR/DNA transposons. **(C)** Density scatter plot demonstrates relationship between DNA methylation differences of TEs and their expression differences. **(D)** Pie chart (without annotation of TEs) demonstrates distribution of TEs with DMRs in human genome. **(E)** Calculation of total gene counts adjacent to TEs with hyper-DMRs in healthy and recovery group. The median, first, and third quartiles are shown in boxplots. Two-sided Wilcoxon signed-rank test were used for all the comparisons. **(F)** Scatter plot of the relationship between DNA methylation differences at TEs and expression differences of genes with promoters containing TEs with DMRs. **(G)** Functional enrichment of upregulated/downregulated genes whose promoters contain TEs with hypo/hyper-DMRs by Metascape. **(H)** The upper pie chart demonstrates the distribution of TEs overlapped with DEG`s promoters in TE subfamilies. Enrich score was calculated by (TE subfamily ratio of TEs overlapped with DEG`s promoters)/(TE subfamily ratio of TEs overlapped with all gene`s promoters). The bottom pie chart shows the distribution of DMRs overlapped with gene`s promoters in TE subfamilies. Enrich score was calculated by (TE subfamily ratio of DMRs overlapped with gene`s promoters)/(TE subfamily ratio of gene promoters). Sliding windows was made across gene promoters with 500 bps consistent with our call DMR strategy. Chi-square tests was used for statistics, enrichment score >1 and *p*-value < 0.05 was defined as enrichment. **(I)** UCSC genome browser view of RNA-seq data demonstrated increased SIK1 expression in recovery group and position of hypo-DMR in SIK1 promoter.

Next, we focused on DMRs between healthy and recovery groups within TE loci, and identified 13,233 DMRs. The percentages of these DMRs were as follows: 36.48% in LINE, 38.23% in SINE, 15.71% in LTR, and 9.58% in DNA transposon (Figure 7A). Subfamilies of SINE with altered DNA methylation were mainly Alu, subfamilies of LINE were mainly LINE1, subfamilies of LTR were mainly ERVL-MalR, and subfamilies of DNA transposon were mainly hAT-Charlie (Figure 7B). We then analyzed expression changes of different TEs with altered DNA methylation and identified upregulated expression of TEs (which are mainly localized at introns) associated with both hyper- and hypo-DMRs in TEs (Figure 7C). Upregulation of certain TEs without hypo-DMRs could be explained by aberrant expression of specific transcriptional regulators. DMRs at TE loci are mainly distributed at intergenic regions, intron, and promoter regions (Figure 7D). To explore the potential impact of DNA methylation changes of intergenic TEs on gene expression, we identified adjacent genes of intergenic TEs with altered DNA methylation and calculated total normalized gene counts for each sample. Interestingly, we observed significant upregulation of genes adjacent to TEs with increased DNA methylation, suggesting that these intergenic TEs act as distal gene silencers (Figure 7E). Further analysis revealed that 19 hypo-DMRs annotated at TE loci were located at the promoter of upregulated genes, and nine hyper-DMRs annotated at TE loci were located at the promoter of downregulated genes (Figure 7F). GO analysis showed their function in ERK signaling regulation and T cell activation (Figure 7G). Furthermore, we identified DMRs at promoter regions that were annotated as various TE subfamilies, although most of those TE subfamilies were not more enriched in DMRs at the gene promoter region relative to other regions in the human genome (Figure 7H). One example is the SIK1 gene which was upregulated in the recovery group and its promoter contains hypo-DMR overlapped with SINE (Figure 7I). These results indicated that TEs with altered DNA methylation may function as regulatory elements for adjacent gene expression.

## Discussion

Our previous study on SARS-CoV-2-infected human cell lines showed viral infection-induced gene misregulation and upregulation of TEs (Yin et al., 2021). However, how TEs behave in the human body remain elusive. To identify how the transcriptional program responds to SARS-CoV-2 in the human body, we downloaded and analyzed a public RNA-seq dataset of peripheral blood samples from COVID-19 patients in the acute phase (Bernardes et al., 2020). As anticipated, we observed both misregulation of genes and aberrantly activation of TEs.

Despite extensive investigations, the outcome of SARS-CoV-2 infection, long-term health consequences (Nabavi, 2020), and long-term recovery progress of COVID-19 patients remain elusive. The majority of the COVID-19-related DMRs are near the gene promoter regions and were hypo-methylated even though the global methylation level remains similar between healthy control and COVID-19 patients (Balnis et al., 2021). However, currently reported results on DNA methylome of COVID-19 patients (Bernardes et al., 2020; Balnis et al., 2021) all depended on 450K or 850K methylation array which covered only a small percent of CpG sites (1% 3%) and could not be used to obtain DNA methylation information of transposable elements. Our WGBS data produced nearly whole-genome CpG sites coverage (coverages of all samples are around 80%) which is valuable for genome-wide identification of differentially methylated regions, especially for transposable elements. Here, we asked whether 3 months was enough to restore the transcriptome and DNA methylation of the patient’s peripheral blood cells to normal. Based on our results, even though the global methylation level remains the same between healthy control and 3-months recovered patients, the overall transcriptome or epigenome profile of peripheral blood samples from the recovery group is still sufficiently different from the control group.

Our result shows that genes involved in leukocyte differentiation are enriched in both upregulated and downregulated genes, and this means that leukocyte differentiation was disturbed (Supplementary Table S6). In upregulated genes, we observed the RELB gene which is the key component of the NF-kappa-B complex, and the NF-kappa-B pathway has been reported to play an important role in leukocyte differentiation (Maslova et al., 2020). We also found upregulation of BCL3 and NFKBIZ. BCL3 is a transcriptional activator that promotes the transcription of NF-kappa-B target genes (Bours et al., 1993), and NFKBIZ is involved in the regulation of NF-kappa-B transcription factor complexes (Cowland et al., 2006; Totzke et al., 2006). Notably, we noticed the downregulation of LILRB4 which was reported to interfere with NF-kappa-B upregulation (Chang et al., 2002). Moreover, downregulated genes also include CEBPA and HDAC9 which are coactivators/corepressors and participate in transcriptional regulation to control cell proliferation and differentiation, and they may orchestrate the expression of NF-kappa-B targets in a context-dependent manner. Therefore, some immune response-related biological processes like leukocyte differentiation were severely disturbed instead of being simply activated or inhibited in recovery COVID-19 patients, through pathways such as the NF-kappa-B pathway. For patients in the acute phase and recovery stage, 48 genes mainly involved in immune responses were consistently misregulated, implicating their long-term involvement in cellular responses to SARS-CoV-2. Interestingly, those genes include transmembrane receptors like PILRB, voltage-gated channel protein HVCN1, and transcription factors like BCL6 and NR4A1, etc. PILRB is a cellular signaling activating receptor and is involved in the regulation of the immune system. Upregulation of PILRB in recovery patients indicates its involvement in orchestrating immune cell types. Voltage-gated channel protein HVCN1 is expressed in immune cells and its mRNA level was downregulated in the recovery group. HVCN1 facilitates reactive oxygen species generation in phagocytosis during immune responses and its downregulation might reflect inefficient immune responses upon SARS-CoV-2 infection, leading to an extended time before full recovery. We also detected upregulated transcription factors BCL6 and NR4A1. BCL6 is a zinc finger transcription factor and regulates the transcriptional activity of STAT-dependent IL-4 responses in B cells. NR4A1 is expressed in human lymphocytes and regulates transcription in response to stress stimuli. These two transcription factors might play key roles in controlling the cell fate of immune cells in COVID-19 patients and contribute to long-term COVID-19 symptoms. Notably, sub-families of TEs remained upregulated 3 months after recovery, suggesting that a longer timeframe may be needed to return to normal levels. Retrotransposons can encode proteins and form retrovirus-like particles (Grow et al., 2015), so it is possible that some virus-like particles visualized by electron microscopy (EM) in COVID-19 patients (Yao et al., 2021) may derive from TEs like LTRs due to their enhanced expression rather than SARS-CoV-2.

While some aberrant gene expression can be interpreted by DNA methylation changes, other mechanisms undermining the transcriptional network in the human body need further exploration. Our WGBS analysis showed no significant changes in global DNA methylation and no significant changes in DNA methylation at TE subtypes, and this may be caused by bulk and heterogeneous levels of cells. Interestingly, we still found 18,516 DMRs between the healthy and recovery groups, and 13,233 DMRs were within TE loci. This supported altered TE expression and changes in DNA methylation at TE loci. Next, we conducted PPI network analysis using STRING database to explore gene interaction at protein level. We used significantly downregulated/upregulated genes in RNA-seq result which also overlapped with DNA methylation changes. As shown in Supplementary Figure S6, several genes did have strong interactions. For example, we found that the chemokine receptor CCR7 was downregulated with hyper-methylation at the promoter, and transcription factor RUNX3 was upregulated with hyper-methylation in the gene body in recovery COVID-19 patients. This finding supports that RUNX3 protein was increased to repress mRNA level of CCR7, because it was previously reported that RUNX3 protein inhibits *Ccr7* expression immune cells in mice (Fainaru et al., 2005). It should be noted that there was a contribution of cell population differences between healthy and recovery groups in identified genes/TEs which showed differential expression patterns or DNA methylation patterns. For example, healthy and recovery individuals may have different circulating memory T cells/exhausted T cell features. However, at least part of those changes at TE loci should be due to TE activation in acute and recovered patients. One supporting evidence of TE activation is that our previous study on human cell lines showed upregulation of TE upon SARS-CoV-2 infection (Yin et al., 2021). Another important supporting evidence is that our analysis of RNA-seq data of acute patients showed activation of the cGAS-STING pathway (see Figure 1B for cGAS upregulation in acute patients) which can be triggered by upregulation of retrotransposon-derived cytoplasmic DNA (Decout et al., 2021). Interestingly, a recent preprint manuscript showed that viral load of SARS-CoV-2 was positively correlated with TE upregulation (Sorek et al., 2021), and this is in agreement with our result that TE activation was positively correlated with COVID-19 severity at the recovery stage. However, the study also showed that TE activation was relatively mild during SARS-CoV-2 infection, and this may be partially explained by the cGAS-STING pathway which can be activated upon SARS-CoV-2 infection and may be responsible for the degradation of cytoplasmic retrotransposon-derived DNA to inactivate retrotransposons. We did not identify significant activation of cGAS gene in recovered patients, probably because TE activation is not severe enough to activate the cGAS-STING pathway. Besides transcriptomes and DNA methylomes, whether COVID-19 leaves other irreversible sequelae requires further investigation, such as telomere length (TL) and mitochondrial DNA (mtDNA) copy number, which is associated with many diseases including cardiovascular diseases, psychiatric disorders, cancers, and inflammatory diseases (Shay, 2016; Sun and St John, 2016).

Our study reveals genes with aberrant expression and genomic regions with altered epigenetic modification in COVID-19 patients 3 months after recovery. Our results support the long-term disease stage marked by an overactivated immune response. Besides, this report provides potential genomic targets to facilitate the convalescence of COVID-19 patients. Moreover, we provide potential transcriptional and epigenetic signatures to track SARS-CoV-2 infection history, identify the profound viral impact on human cells and reveal long COVID-19 risks. However, due to genetic mutations of SARS-CoV-2 variants, gender, health, age of patients, and other factors, transcriptional and epigenetic changes may vary and need further validation and investigations.

In summary, we examined the transcriptomes and DNA methylomes of COVID-19 patients 3 months after recovery, and noticed that both genes and TEs were impacted at transcription and DNA methylation levels. Misregulated genes were involved in immune response and other biological functions, such as stress response, and metabolic processes, while TEs in intron and other regions were specifically activated which may disrupt the transcription process and genome integrity and induce inflammation. Furthermore, DNA methylome analysis showed that genes with DMRs were also involved in immune response-related processes; and differentially methylated promoter and distal intergenic region may play important roles in gene regulation. Finally, altered CG methylation can indirectly impact gene expression and may play regulatory roles in stress, illness, and aging. Further studies are needed to track changes in transcriptome and DNA methylome of COVID-19 patients for a longer time to identify how long is required for a full recovery.

## Data Availability

The datasets presented in this study can be found in online repositories. The names of the repository/repositories and accession number(s) can be found below: Bioproject PRJCA006301, https://ngdc.cncb.ac.cn/bioproject/browse/PRJCA006301.

## Abbreviations

WGBS, whole-genome bisulfite sequencing; DMR, differentially methylated region; TEs, transposable elements; LINEs, long interspersed nuclear elements; SINEs, short interspersed nuclear elements; LTRs, long terminal repeats; DNAs, DNA transposons; DEGs, differentially expressed genes; DETEs, differentially expressed TEs.
